# Two dipeptide repeat proteins are produced from mammalian telomeric RNA previously thought to be a long non-coding RNA

**DOI:** 10.1080/15476286.2026.2705075

**Published:** 2026-07-20

**Authors:** Taghreed M. Al-Turki, Jack D. Griffith

**Affiliations:** Lineberger Comprehensive Cancer Center, Departments of Microbiology and Immunology, and Biochemistry and Biophysics. University of North Carolina at Chapel Hill, Chapel Hill, NC, USA

**Keywords:** Telomeres, TERRA, Repeat-associated non-AUG (RAN) translation, GL dipeptides, VR peptides

## Abstract

Studies of neurological diseases caused by the expansion of nucleotide repeats led to the discovery that RNA can undergo translation by ribosomes in the absence of canonical AUG start signals, a process termed repeat-associated non-ATG translation (RAN). This discovery suggested that RNA transcribed from mammalian telomeres, termed TERRA, could generate RAN products. Indeed, two dipeptide repeat proteins can be produced: repeating arginine–valine (VR) and repeating glycine–leucine (GL). Both VR and GL form amyloid aggregates, and VR was observed to be expressed in cells with elevated TERRA, including a human osteosarcoma line. VR undergoes a change in aggregation state during mitosis, where it becomes dispersed, binds ribosomes and can depress translation, possibly playing a regulatory role in the cell cycle. The discovery that RAN translation can occur on telomeric RNAs has opened new connections between telomeres, ageing and the generation of RAN proteins with important biological activities.

## RAN translation and its discovery

Repeat-associated non-AUG (RAN) translation was first reported in 2011 [1] from studies on spinocerebellar ataxia type 8. It refers to protein synthesis that can occur without the requirement of a canonical AUG, often on structured repeat RNAs, and which generated products across multiple reading frames from both sense or antisense RNAs [1]. The discovery of RAN translation originated from studies of two CTG:CAG repeat expansion disorders caused by expansions in different genes: spinocerebellar ataxia type 8 (SCA8; *ATXN8OS*) and myotonic dystrophy type 1 (DM1; *DMPK*). It was observed that three homopolymers – polyglycine, polyserine and polyalanine – were produced due to the triplet expansion. Notably, although the CTG:CAG expansions in SCA8 and DM1 both generate polyalanine via RAN translation, the resulting proteins are not identical because the repeat tract is embedded within different transcript contexts, producing different C-terminal sequences. Surprisingly, experiments with plasmids carrying the CTG expansion but lacking the ATG start codon still produced these poly amino acids in cells. Furthermore, translation was observed in cell culture with hairpin-forming CAG but not with non-hairpin CAA repeats [1]. The model proposed was that the base-paired hairpins formed by the CAG repeats allow ribosomes to bind and initiate translation, whereas CAA repeats do not pair and thus do not provide sites for ribosome loading [1]. The accumulated RAN translation products have also been detected *in vivo* in mouse tissue and human patient samples from autopsy brain [1]. Together, these findings established that structured repeat RNAs can support non-canonical translation and generate distinct repeat proteins in multiple frames, linking repeat sequence and RNA structure to protein output [1]. This conceptual advance immediately raised the possibility that other disease-associated repeat expansions might also produce toxic proteins via similar mechanisms. Shortly thereafter, the discovery of the C9orf72 G4C2 expansion in inherited amyotrophic lateral sclerosis/frontotemporal dementia (ALS/FTD) provided a major new context for testing these ideas.

In 2011, a large GGGGCC (G4C2) hexanucleotide repeat expansion in intron 1 of the C9orf72 gene was identified as the most common cause of inherited ALS/FTD [2,3]. While unaffected individuals exhibit fewer than 23 G4C2 repeats at this locus, patients with ALS/FTD exhibit expansions ranging from hundreds to over 1000 repeats. RAN translation from the C9orf72 repeat expansion occurs in three reading frames from each of the sense (G4C2) and antisense (G2C4) transcripts, producing six distinct dipeptide repeat proteins (DPRs) in total: from the sense strand, glycine–alanine (GA), glycine–proline (GP) and the highly charged glycine–arginine (GR); and from the antisense strand, proline–alanine (PA), the highly charged proline–arginine (PR), and a second glycine–proline species. Although the two GP proteins share the same internal dipeptide repeat, they differ in their C-terminal sequences [4]. Antibodies recognizing these DPRs identified their presence in inclusions within brain autopsy samples, supporting the role of RAN translation in the pathology and toxicity of ALS/FTD. Because mammalian telomeres are also transcribed into hexameric G-rich repeat-containing RNAs (TERRA) [5,6], it was proposed that TERRA might also be a substrate for non-AUG translation and repeat product generation [7].

## RAN translation diverges from canonical initiation by using non-canonical start-site selection (non-AUG) and can increase under cellular stress

In canonical mRNA translation, initiation is cap-dependent, in which eIF4E within the eIF4F complex binds the 5′ m^7^ G cap, eIF4G bridges to eIF3 to recruit the 43S pre-initiation complex (PIC) that carries 40S-eIF2 tRNAi-Met. After assembly, eIF4A, stimulated by eIF4B/H unwinds the 5′-end as the PIC scans to the first AUG start codon. Start-site selection triggers factor release, and 60S ribosome subunit joining to form the 80S ribosome for elongation. In contrast, in cells, RAN translation has been reported to initiate without a canonical AUG, often via non-AUG or near-cognate start sites (e.g. CUG) and/or within repeat contexts [1,8,9]. Notably, rabbit reticulocyte lysates, one of the most commonly used cell-free translation systems, is often less permissive for AUG-independent initiation and frequently shows stronger dependence on an AUG or near-cognate start codon for robust translation. Therefore, cell-free assays are best used to probe potential initiation mechanisms, but they may not capture AUG-independent initiation as it occurs *in vivo* [1,10,11].

At the transcript level, initiation-site choice reflects both local sequence context and RNA secondary structure within repeat regions [12]. In the Fragile X-associated tremor/ataxia syndrome (CGGn triplet expansion) for instance, C–G pairing in the RNA produces stable duplex hairpin structures that slow cap-dependent scanning, increasing transient stalling just upstream of the repeat and thereby making near-cognate start codons (e.g. CUG, GUG) more likely to be selected. As such RAN initiation was suggested to most often occur from these upstream sites, as shown using CGG-repeat reporter constructs in mammalian cell lines and *Drosophila* models of FXTAS [13,14]. In C9orf72 (G4C2 repeats), initiation can occur at an upstream near-AUG or within the repeat itself, and both cap-dependent and cap-independent modes have been reported, based on studies using G4C2 reporter cell lines and patient-derived neurons [12].

RAN protein expression can increase under cellular stress, including activation of the integrated stress response (ISR), which phosphorylates eIF2α and can relax start-site stringency, making near-cognate initiation more competitive [9,15]. One upstream route to ISR-like signalling in C9orf72 models involves PKR (protein kinase R; EIF2AK2), a dsRNA-activated eIF2α kinase. During the ISR, elF2a reduces canonical initiation and can increase the competitiveness of near-cognate start sites; consistent with this, C9orf72 RAN translation is upregulated by stress via eIF2α phosphorylation [9,15]. In C9orf72 BAC mice, inhibiting PKR either by expressing a dominant-negative PKR construct or treatment with the drug metformin reduces RAN/DPR protein accumulation and improves disease-relevant phenotype [16]. When the eIF2–GTP–Met–tRNAi complex becomes limiting, alternative initiation and reinitiation pathways can help sustain translation initiation, and several non-canonical factors, most notably DENR/MCTS1 and, in some settings, eIF2A, have been shown to modulate CGG and G4C2 RAN translation [17]. These mechanisms determine the quantity and type of DPRs produced from repeat transcripts, setting the stage for understanding how specific DPR species drive cellular toxicity and neurodegeneration.

## C9orf72 DPRs contribute to cellular toxicity and neurodegenerative phenotypes

The C9orf72 DPRs have been shown to contribute to cellular toxicity and neurodegenerative phenotypes. Overexpression of G4C2 repeat expansions from plasmids in cell culture is sufficient to induce toxicity. Among the most harmful DPRs are the arginine-rich dipeptides GR and PR, which frequently accumulate in the nucleus/nucleolus and perturb RNA-related processes and translation. In contrast, poly(GA) is notable for forming prominent cytoplasmic aggregates that are often ubiquitin/p62-positive and linked to impaired proteostasis, including increased burden on the ubiquitin–proteasome system [18–20]. Studies in cultured cells have also shown that GR and PR, when expressed from plasmids, compromise mitochondrial function, increase oxidative stress, cause a severe ribosomal stalling [21–29] and form toxic nuclear aggregates that result in neuronal death [27]. Additionally, PR has been observed to localize to nucleoli, where it disrupts RNA biogenesis [30]. In the latter context, Loveland et al [31] examined the interactions of PR and GR with ribosomes, observing that when either peptide consisted of 20 repeats or more, translation in an *in vitro* firefly translation system was repressed. Their high‑resolution cryoEM structure of PR with ribosomes revealed that the peptide bound tightly to the exit tunnel in the large ribosome subunit where it would effectively block translation.

In animal models, these toxic effects extend to complex neurodegenerative outcomes, but phenotypes vary by model class and expression strategy. In an AAV-mediated somatic CNS overexpression model of expanded G4C2 repeats, mice develop RNA foci and DPR accumulation along with TDP-43 pathology, neuronal loss and behavioural deficits [32].

Using a C9orf72 BAC transgenic approach, Liu et al. generated mice carrying the full-length human C9orf72 gene with a large repeat expansion. These mice developed sense and antisense RNA foci, DPR accumulation, TDP-43 pathology, motor-neuron and cortical and hippocampal degeneration, muscle denervation and progressive motor deficits [33]. These models have enabled the testing of whether reducing DPR production or DPR burden can mitigate disease phenotypes *in vivo*. DPR-directed immunotherapy improves C9orf72-associated phenotypes in BAC transgenic mice [34]. Inhibition of the PKR pathway, either by expressing a dominant-negative PKR construct or by metformin treatment, which the authors show suppresses PKR pathway activation, reduces DPR accumulation and improves phenotypes in C9orf72 BAC transgenic mice [16]. Finally, blocking RAN translation in an AAV model without altering repeat RNAs improved C9orf72-related ALS/FTD outcomes [35]. Similarly, in Zebrafish models, overexpression of long G4C2 repeats results in notable motor axon abnormalities, including impaired axonal growth and irregular branching, while shorter repeats (less than 10) failed to induce the same effects [36,37]. Moreover, studies in Drosophila melanogaster have demonstrated that the expression of GR and PR results in retinal degeneration and morphological deterioration in motor neurons, further supporting the toxic role of these DPRs in neurodegenerative processes [38], many likely linked to disruption of normal RNA function and trafficking.

## C9orf72 DPRs generate phase separation droplet-like bodies in cells

Studies of the C9orf72 DPRs, especially PR and GR have pointed to their involvement in phase separation, a process essential for the formation of membrane-less organelles and found to be diagnostic of ALS/FTD pathology [39]. GR and PR facilitate phase separation through interactions with RNA and other cellular components [4]. These DPRs form distinct cytoplasmic inclusions with liquid-like characteristics, including droplet formation [4]. The properties of the droplets, including size and viscosity, are also dependent on the type and structure of the RNA involved in droplet formation [40]. Post-translational modifications, such as methylation, also impact the behaviour of the DPRs in phase separation. Methylation of GR, for example, reduces its tendency for phase separation and decreases its toxicity, potentially offering a protective effect in disease contexts [41].

The toxicity associated with droplets formed by C9orf72-derived DPRs, particularly GR and PR, arises from their capacity to sequester critical cellular components, disrupt essential cellular functions and provoke sustained cellular stress responses. These liquid-like droplets can selectively sequester key biomolecules, notably RNA-binding proteins and other factors crucial to transcription, translation and RNA metabolism [4,42,43]. As a consequence, these essential cellular components are prevented from performing their physiological roles, thereby significantly disturbing cellular homoeostasis and eliciting stress-related pathways [44]. Collectively, while physiological phase separation serves as a critical mechanism for cellular organization, the aberrant formation of DPR-induced droplets represents a pathological event leading to severe cellular impairment.

## RAN mechanisms in the context of telomeric RNA

Located at chromosome ends, telomeres serve as crucial protectors of genomic stability, blocking chromosomes from joining end-to-end. The telomeric DNA–protein complex consists of a long array of short nucleotide repeats (TTAGGG in mammals) bound by general chromatin proteins, including the histones and DNA repair factors, and a specialized protein complex known as shelterin. This complex, composed of six core proteins (TRF1, TRF2, POT1, TIN2, TPP1 and RAP1), helps regulate its structure [45]. At their termini, telomeres feature a 3’ single-stranded (ss) overhang of approximately 300 nucleotides. In concert with the shelterin complex, telomeric double‑stranded DNA (dsDNA) can fold back on itself and insert the ss overhang back into the preceding telomeric duplex DNA to generate a looped structure, termed a T-loop [46–48]. These loops are essential for maintaining chromosome integrity, as they protect the chromosome ends from being recognized as damaged DNA, thus preventing end-to-end fusion and degradation. The T-loop structure essentially ‘hides’ the chromosome ends, shielding them from DNA repair mechanisms that might otherwise mistake them for breaks [48,49].

In addition to blocking chromosome ends from fusion, it was known that the length of the telomeric DNA is reduced by roughly the size of an Okazaki fragment during each round of replication [45,50]. This progressive shortening triggers cellular senescence pathways once a critical telomere length has been reached [51,52]. Thus, telomeres were recognized as ‘cellular aging clocks’, limiting cellular lifespan and blocking cells from unlimited replication [53]. Hence, it was assumed that the sole function of telomeric DNA is to limit the number of cell divisions and thus generate an ageing clock. However, this perspective shifted dramatically in 2007 when it was discovered that mammalian telomeres are transcribed from sub-telomeric Pol II promoters with the transcripts continuing into the telomeric dsDNA repeats and ranging from 100 to over 9000 nt [5,6]. The predominant transcript is from the C-rich telomeric strand, giving rise to a G-rich RNA termed TERRA (Telomeric Repeat-containing RNA). This discovery revealed that telomeres are not silent and can generate long RNAs that play active roles in regulating telomere length and function.

TERRA transcripts carry a 5′ m^7^ G cap and exist as two major fractions: a chromatin-bound, non-polyadenylated fraction and a more soluble nucleoplasmic/cytoplasmic poly(A)+ fraction that accounts for roughly 40% of total TERRA in human cells [54–56]. Poly(A)− TERRA is preferentially enriched at telomeric chromatin, whereas poly(A)+ TERRA is in the nucleoplasm and cytoplasm, consistent with distinct functional compartments. Under osmotic or oxidative stress, the cytoplasmic fraction of TERRA increases in human ALT cells, with 20% found colocalized with the stress granule (SG) protein marker G3BP1 [57]. Importantly, SGs are membraneless cytoplasmic ribonucleoprotein condensates that assemble when translation initiation is globally inhibited; they are enriched in non-translating messenger ribonucleoprotein complexes (mRNPs) and contain 40S ribosomal subunits and multiple initiation factors, consistent with accumulation of stalled pre-initiation complexes rather than ongoing bulk translation. In this context, SG association can place TERRA in an initiation-factor-rich environment during stress, but colocalization alone does not demonstrate active non-canonical initiation within SGs. Supporting the notion that repeat-rich RNAs can interact with stress granules, overexpression expanded G4C2 RNA has been reported to promote RNA-granule condensation and to induce TIAR-positive stress granules in cellular models [58,59].

Consistent with a potential link between cytoplasmic TERRA and translation machinery under stress, the TERRA-mimicking oligo used by Larini et al. [57] pulled down a broad set of translation–initiation proteins from a cytosolic lysate, including multiple eIF3 subunits (A–M), eIF4A1, eIF4B, eIF4G1/2 (DAP5/eIF4G2), eIF4E2 (4EHP), DDX3X, DHX29, eIF2/eIF2B subunits, and several 40S ribosomal proteins. Together with prior work implicating stress adaptive initiation routes, this interactome points to engagement of cap-dependent scanning as well as eIF4E-independent initiation modules (e.g. eIF3d- or DAP5/eIF4G2-linked mechanisms) during cellular stress [12,60]. Telomere stress itself can elevate telomere-derived repeat RNA: oxidative stress induces TERRA, and telomere deprotection (for example, TRF2 loss) drives broad TERRA upregulation [61,62]. In severe telomere dysfunction during replicative crisis, telomere-derived TERRA can engage cytoplasmic signalling via mitochondrial TERRA–ZBP1 complexes, and TERRA has also been reported to localize to stress granules—together expanding the repeat-rich RNA substrate available for stress-biased, non-AUG initiation [57,63,64].

More broadly, potential TERRA translation via the RAN mechanism can be viewed as stress-dependent with two steps: (i) access; telomere dysfunction and cellular stress increase export of a poly(A)+ TERRA fraction and promote its accumulation into or near SGs, bringing it into the cytoplasmic environment where non-canonical initiation factors are concentrated [63], and (ii) permission, via initiation-factor engagement (shown by the TeloG pulldown [57]; and mass spec [65] plus DHX36-mediated RNA G4 resolution that enables near-cognate scanning, the same combination of conditions that enhances non-AUG translation at G-rich repeats in other RNAs [12,60,66,67]. Adding the broader RAN rules, RPS25 dependence, eIF4A, B and H requirement, ISR/alternative initiators, ribosome queuing and RQC activity provides a practical checklist for when TERRA is most likely to yield the novel dipeptides VR and GL [7,65].

The possibility that TERRA could undergo non-canonical translation opened a broad new perspective on telomeres, suggesting that they may be considered active cellular organelles, with multiple dynamic roles in the cell cycle. As described below, two DPR proteins, repeating valine–arginine (VR) and repeating glycine–leucine (GL) are encoded by TERRA, and compelling evidence for the presence and function for VR in cells has been obtained.

## Discovery of the telomere DPRs

**Figure 1.**
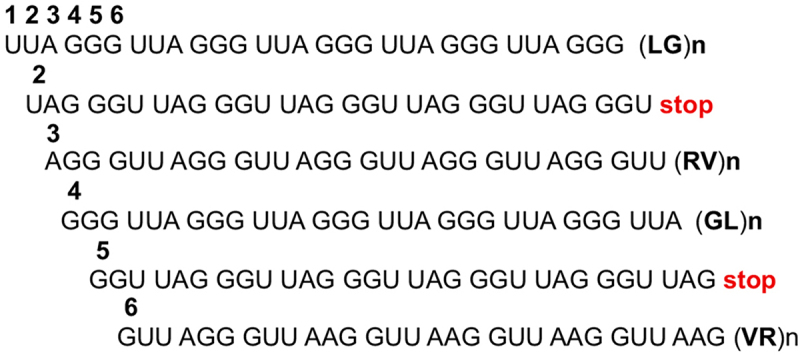
Illustration of RAN initiation on TERRA. Depending on which nucleotide on TERRA RNA is used for initiation of protein synthesis, two different repeating dipeptide proteins are produced. Two stop codons are encountered, and repeating GL and GL are identical as are VR and RV.

The discovery of the telomeric DPRs laid out in 2023 by Al-Turki and Griffith [7] was based on the striking similarity between the 6 nt repeat in the expanded ALS/FTD RNA (GGGGCC)n and TERRA (UUAGGG)n, which is also 6 nt long and G-rich. RAN translation of TERRA would produce two DPRs. One, repeating glycine–leucine (GL), is very hydrophobic and similar to GA, GP and PA, and the other, repeating valine–arginine (VR), closely resembles PR and GR. These striking similarities lighted a pathway in designing experiments to test the possible presence and properties of the two telomeric DPRs in cells. A diagram of how RAN translation using TERRA as a template would generate VR and GL is shown in Figure 1. Of note, the telomeric repeat of *C. elegans* is TTAGGC, and analysis as in Figure 1 revealed that this repeat also has the capacity to generate two DPRs: GL, the same as mammalian GL, and LR, which is similar to VR, GR and PR. Staining *C. elegans* with an antibody raised against repeating LR suggests that it may be expressed in germ granules (unpublished studies, Shawn Ahmed laboratory). Analysis of potential RAN translation of TERRA from plants that employ TTTAGGG repeats revealed stop codons in all frames. While the work to date has focused on DPRs that consist of pure dipeptide repeats, *in vivo*, variations in the telomeric repeats, for example, small insertions or deletions, could generate TERRA molecules in which RAN translation would begin, for example, with a run of VR repeats but then continue with GL repeats, generating a family of DPRs with varied biological properties. However, since both pure VR and pure GL form amyloid filaments, one would assume that a mixed DPR would also do so.

**Figure 2.**
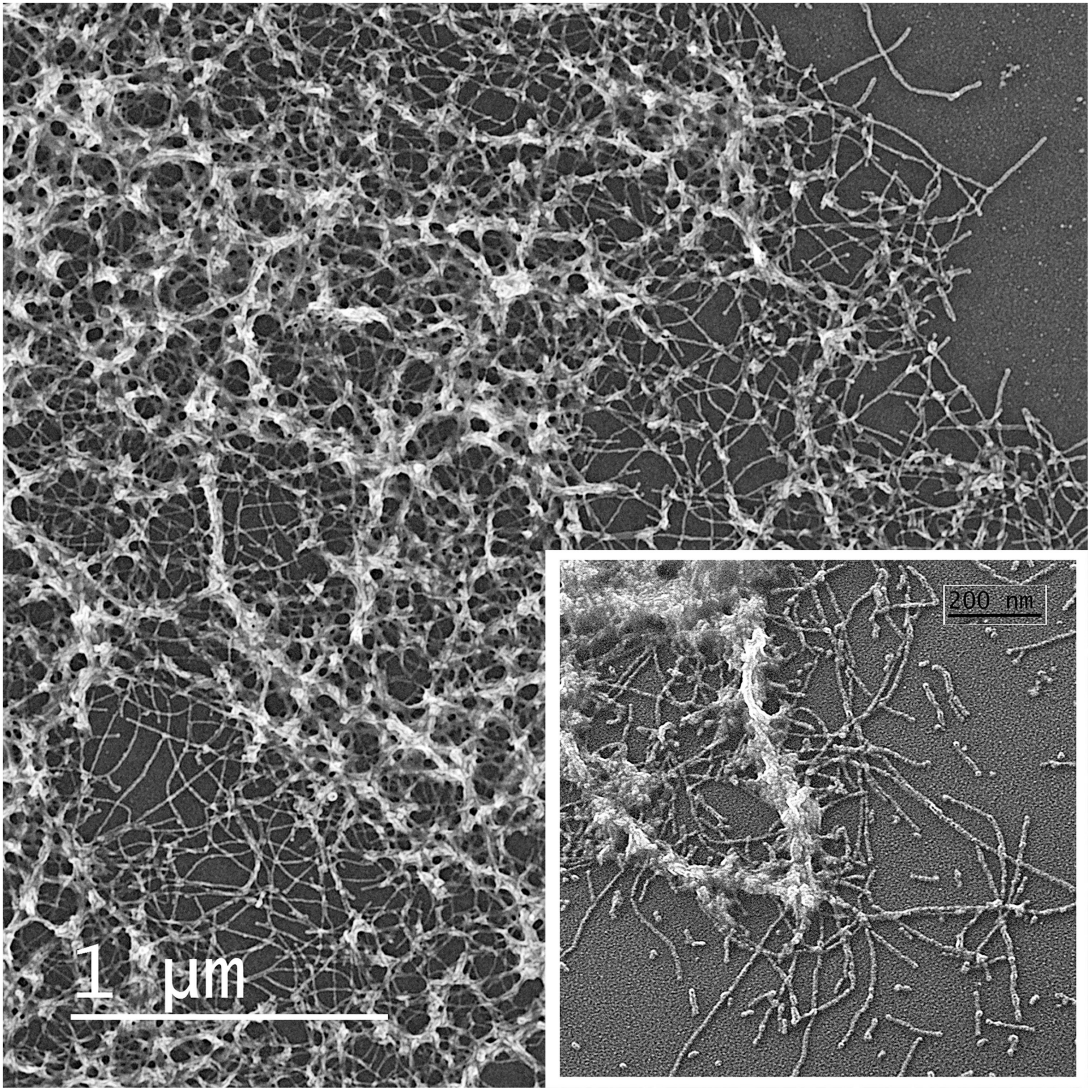
Filaments and amyloids formed by the GL dipeptide repeat protein. Repeating GL dipeptide protein (18 amino acids in length) was chemically synthesized and taken up in 10 mM Tris–HCl pH 7.5, 1 mM EDTA buffer at 20 µg/ml and allowed to rapidly form 10 nm diameter filaments which then aggregated into amyloid mats. Sample was prepared for EM by mounting onto thin carbon foils, dehydration and rotary shadow casting with tungsten. Shown in reverse contrast. Inset shows higher magnification view.

The possibility that RAN translation could generate VR and GL species in cells led us to have these peptides chemically synthesized in lengths from 8 to 40 amino acids. Electron microscopic (EM) examination of solutions of each revealed that both can form 10–12 nm diameter long fibres, which rapidly associate into large amyloid networks. The image of a GL amyloid network in Figure 2 shows striking similarity to images of ones formed by GA from ALS/FTD studies [7,68,69]. The similarity between GL and C9orf72-derived GA fibrils suggests that amyloid-like self-assembly may represent a common biophysical property of repetitive peptides generated through distinct biological mechanisms. Whether these assemblies serve physiological functions, contribute to pathology or both likely depends on their cellular context and abundance. In C9orf72 diseases, GA and PR have been found to activate innate immune pathways such as the NLRP3 inflammasome and cGAS-STING signalling [70–72]; thus, we speculate that telomeric dipeptides at certain levels may activate inflammatory pathways.

**Figure 3.**
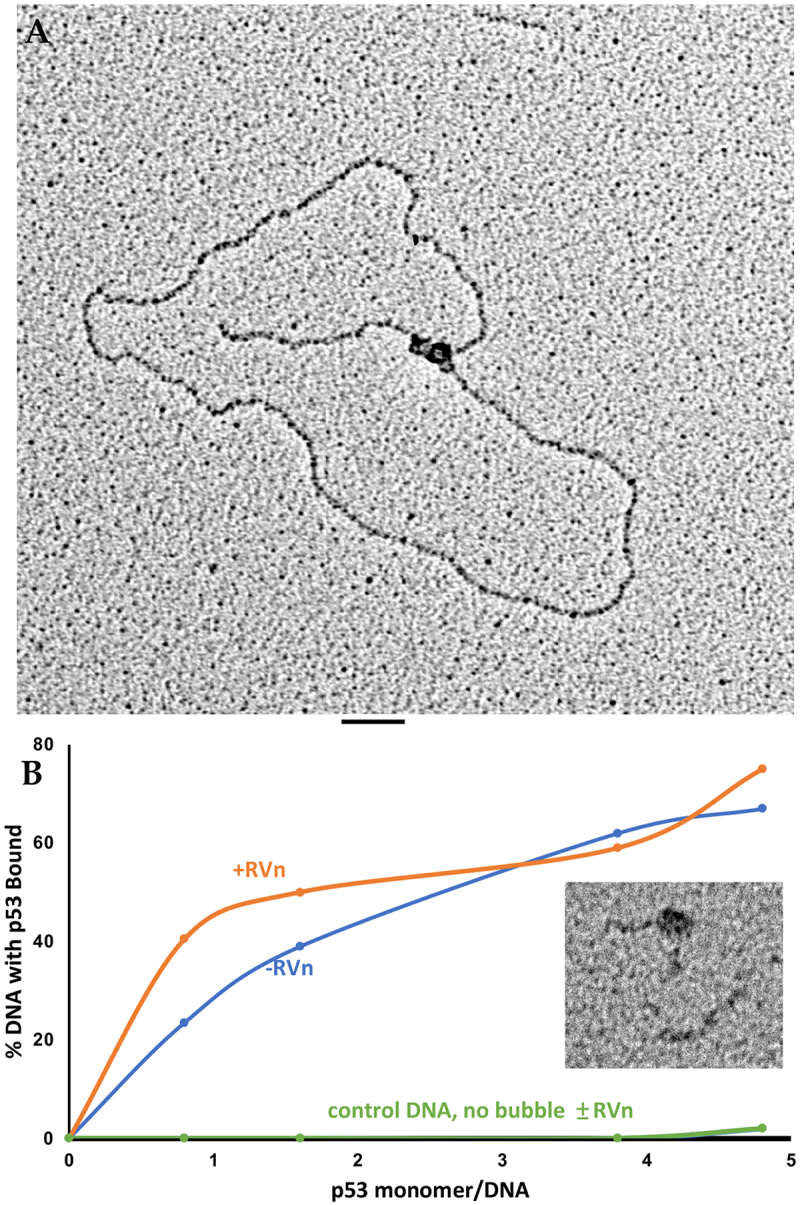
VR dipeptide protein binds DNA and loads p53 onto single‑strand bubbles. (A): VR dipeptide protein was shown in Al Turki and Griffith (2023) to bind tightly to single-strand segments in DNA. In this example, biotin tagged VR_15_ was incubated with a circular DNA plasmid containing a 400 bp displaced fork containing a 15-nt single-strand gap at the base of the displaced. This was followed by addition of streptavidin protein to reveal the location of the bound VR. Greater than 85% of the VR localized to the single-strand gap. (B): A 200 bp duplex containing a 10-nt single-strand bubble at the centre was incubated with p53 protein in the presence or absence of VR_15_ and the fraction of DNAs containing p53 at the centre was scored by EM. Preparation of the samples for EM was as described in Al Turki and Griffith (2023).

Given the high arginine content of VR, it seemed likely it would show an affinity for DNA. Direct EM experiments using ssDNA, dsDNA, and TERRA [7] showed that VR has a high affinity for ssDNA and RNA, rapidly collapsing both at moderate VR concentrations, while dsDNA remained relatively unaffected until high concentrations were added. The high affinity for ssDNA suggested that VR might localize to ssDNA gaps at replication forks. To investigate this, a circular plasmid containing a 400 bp displaced arm with a 15 nt ssDNA gap at the base of the fork was incubated with a VR_20_ peptide containing a biotin tag at the C-terminus. The presence of VR on the DNA was in revealed by adding streptavidin, which is easily seen in the EM. These studies showed that VR has a high specificity for the ss gap with little or no VR bound along the length of the dsDNA (Figure 3(A)).

Further unpublished studies probed the ability of VR to interact with p53 at a lesion in DNA consisting of a looped-out 10 nt ssDNA bubble. While it seemed possible that VR might inhibit p53 binding, the opposite was observed. In the presence of VR, twice as many ss bubbles were bound by p53 as in its absence at the specific DNA to protein ratios shown (Figure 3(B)). The biological ramifications of these observations are significant. In cells undergoing telomere crisis due to short telomeres, telomere damage or mutations in the shelterin proteins, the normal protective T-loop structure is lost or compromised, leading to the release of more TERRA from the chromosomes, where RAN translation will generate elevated levels of VR and GL. VR could then bind replication forks, unpaired Holliday junctions and possibly transcription bubbles, leading to general genomic instability. If this also facilitates p53 loading onto bubbles and forks in DNA, this will both enhance genomic instability and sequester p53 away from its needed roles, leading to a p53 null status. In some ways, this may resemble the bacterial SOS response, in which an insult to the bacterial genome results in the activation of error-prone replication pathways as a potential means of generating new useful genetic arrangements.

## The VR dipeptide repeat proteins are present in human cells, and their levels are affected by stress

An experimental breakthrough was provided by the generation of polyclonal antibodies to repeating VR peptides. To validate their specificity, U2OS cells were transfected with a plasmid encoding 60 VR repeats fused to a FLAG epitope. Following fixation, cells were co-stained with anti-FLAG and anti-VR antibodies and analysed by laser scanning confocal microscopy. Strong colocalization of VR and FLAG signals confirmed the specificity of the VR antibody preparations. This specificity was further confirmed by western blotting, where lysates from VR_60_-FLAG-expressing U2OS cells exhibited bright bands in the gel; the control experiments in cells not expressing the VR_60_/FLAG construct showed minimal background staining, further confirming the antibody’s specificity. Generation of antibodies to GL has so far not been successful.

**Figure 4.**
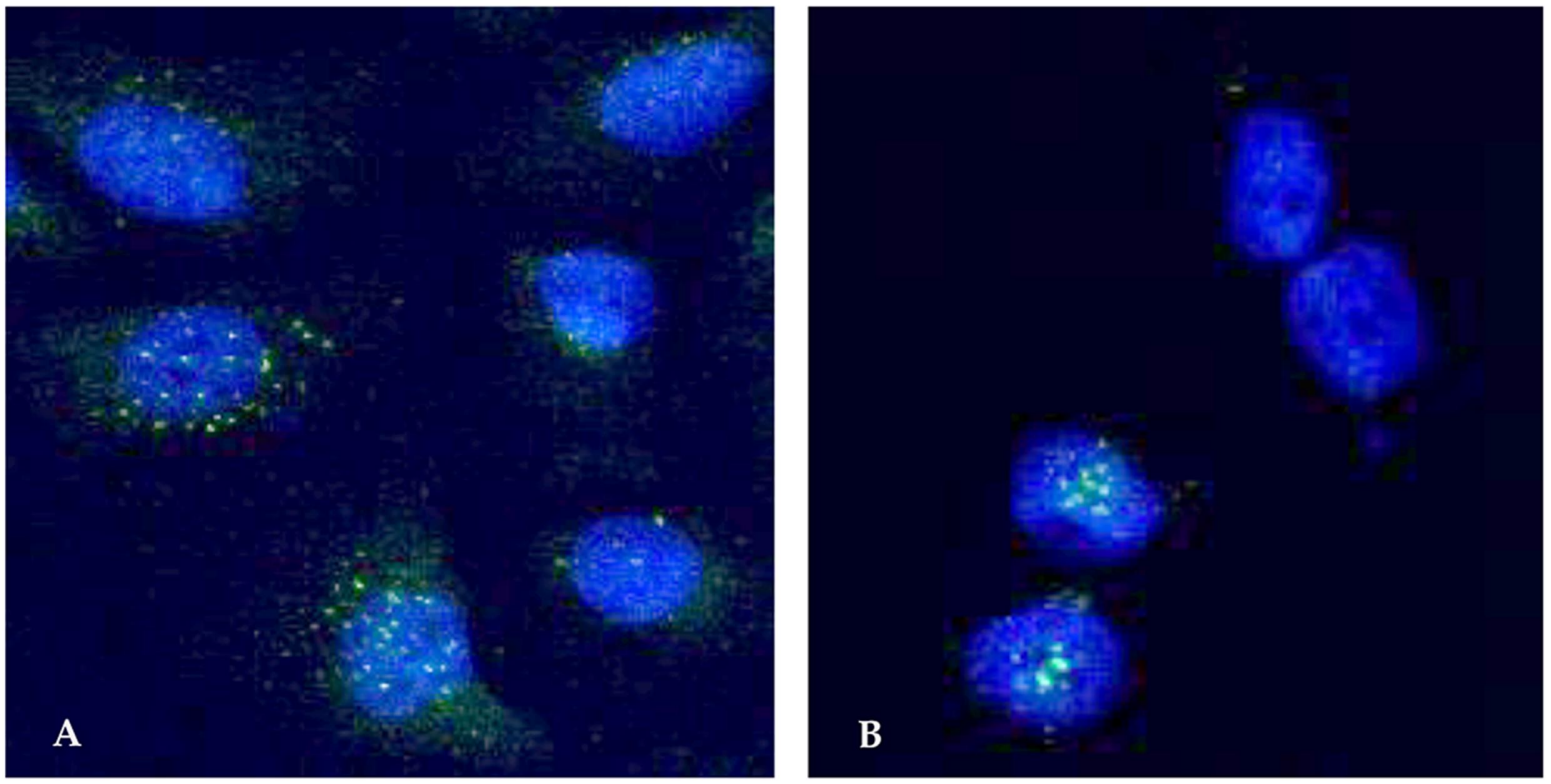
Characterization of VR peptides in U2OS (ALT). Representative confocal images showing nuclei (DAPI-blue), nuclear and cytoplasmic VR aggregates heterogeneous in size and intensity (green). Cells were fixed in 4% PFA and stained with a rabbit polyclonal anti-VR antibody and Alexa Fluor 488.

Using the polyclonal antibodies to VR, quantitative laser confocal scanning microscopy analysis was utilized to ask if VR could be detected in three different cell model lines. U2OS cells (ALT-positive) and ICF syndrome fibroblasts are both characterized by elevated TERRA levels [73–76] while primary human foreskin fibroblasts (FSK) cells show lower levels of TERRA expression. Using a vigorous scoring protocol with the light microscopic images, 37% of U2OS cells and 33% of the ICF fibroblasts reached a scoring threshold for punctate VR bodies while just 11% of the FSK fibroblasts did so. Thus, cells with high endogenous TERRA transcription (U2OS and IDF) contained about three-fold more VR positive nuclei than primary FSK cells. Localization analysis was conducted on VR positive cells showing that the majority of the aggregates were nuclear rather than cytoplasmic. Morphologically, VR signals appeared as small punctate speckles or larger spherical condensates, consistent with the behaviour of arginine-rich dipeptides in other systems. Representative confocal images illustrating these findings in U2OS cells are shown in Figure 4.

**Figure 5.**
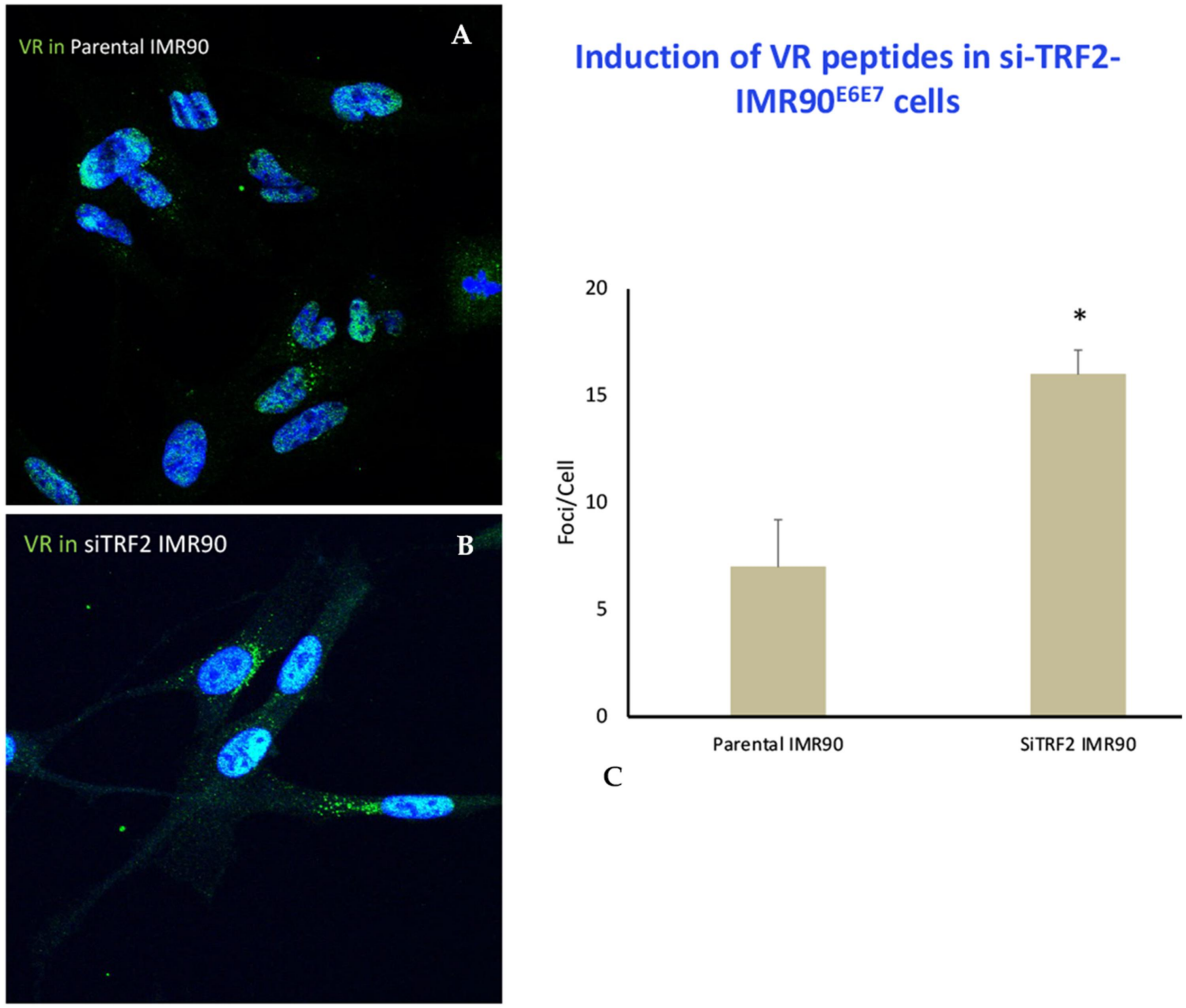
TRF2 knockdown increases VR aggregates in siTRF2-IMR90^E6E7 cells. (A) Representative confocal images of parental IMR90^E6E7 cells immunolabeled with VR primary antibody (green); nuclei were stained with DAPI (blue). (B) Representative confocal images of siTRF2-IMR90^E6E7 cells immunolabeled with VR primary antibody (green) and counterstained with DAPI (blue). (C) Quantification of VR aggregates per cell in parental IMR90^E6E7 and siTRF2-IMR90^E6E7 cells. Data are presented as mean ± SEM from three independent experiments. Two-tailed, unpaired *t* test, *p* < 0.05.

TERRA is expressed from multiple chromosomes and is a required component of chromosome stability, and the highest reductions of TERRA in cells have been in the range of 40% [77]. This results in partial loss of chromosome integrity and additional TERRA translocated to the cytoplasm [63] where it can generate more VR and GL. Indeed, this was observed using an LNA Gapmer to reduce TERRA [7]. LNA treatment of U2OS cells resulted in the appearance of large nuclear condensates staining brightly for VR suggestive of stress-induced phase transitions. These results were further supported using a lentivirus to knock down TRF2 as an additional means of generating dysfunctional telomeres [78]. Expression of this lentivirus in IMR90 cells (Figure 5) produced higher VR levels in the cells and a marked increase in cytoplasmic VR aggregates, reinforcing the link between telomere dysfunction and DPR mis-localization.

**Figure 6.**
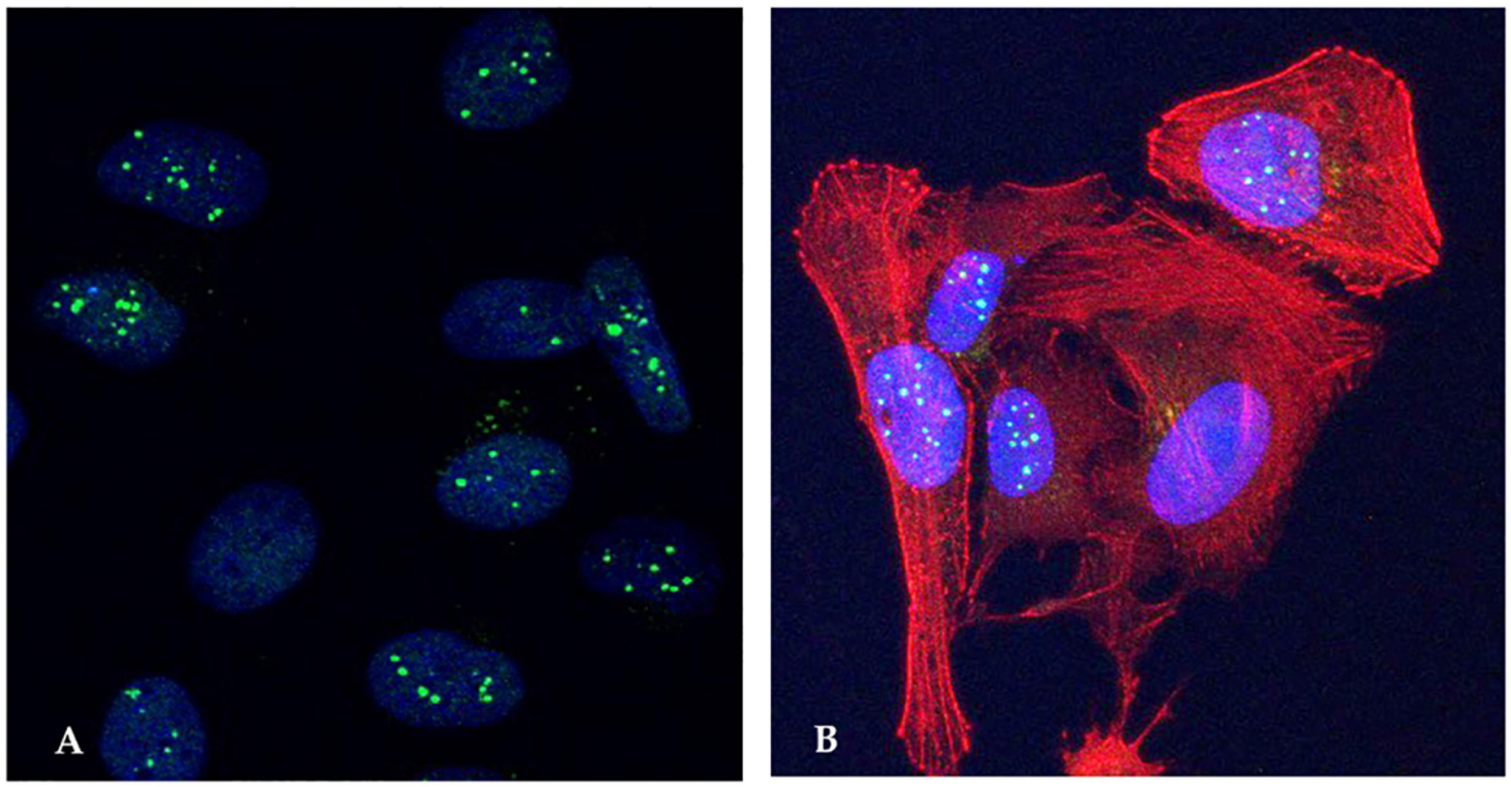
BRACO19-treated U2OS cells display prominent nuclear VR aggregates. U2OS cells were treated with 2 μM BRACO-19 for 24 h, fixed and stained for DNA (DAPI, blue), VR (rabbit anti-VR primary and secondary antibody Alexa Fluor 488, green) and actin (phalloidin, red; right panel). Representative fluorescence images are shown (left, DAPI and VR; right, merged DAPI, VR and phalloidin.

To further probe the link between induction of unstable chromosomes and the appearance of VR deposits in the cells, an acridine derivative, BRACO-19, which has been shown to bind tightly to the G-rich 3’ single-strand overhangs in telomeres [79], was incubated with the U2OS cells. Detailed studies by Neidle and colleagues had demonstrated that cells expressing the ALT phenotype, such as U2OS cells, or ones with very short unstable telomeres, were much more susceptible to BRACO-19 killing than primary cells [80]. This toxicity appears to be due to the tight binding of the drug to the G-quartets in the 3’ overhang, disrupting T-loop structure and rendering telomeres unstable. Incubation of the U2OS cells with 2 µM BRACO-19 for 24 hr followed by staining with the VR antibody and laser scanning microscopy, revealed that a large fraction of the cells had developed large nuclear aggregates appearing typical of liquid droplets in the nuclei (Figure 6).

In summary, telomere damage, exemplified by TRF2 depletion, elicits profound cellular instability by activating DNA damage responses. The observed accumulation of VR peptides under these conditions may exacerbate genomic instability through strong interactions with nucleic acids and replication intermediates, potentially disrupting DNA replication, repair and transcription. Analogous toxic mechanisms have been reported for arginine-rich DPRs (e.g. PR, GR) in ALS/FTD, where aberrant aggregation perturbs nucleocytoplasmic transport, induces neurodegeneration and accelerates age-related toxicity [23,81,82]. These findings highlight VR peptides as potential mediators of telomere-driven cellular dysfunction, suggesting shared pathological mechanisms across DPR-associated diseases and implicating telomeric DPRs in ageing and age-related disorders.

## VR may down-regulate translation at mitosis

The parallels between the ALS/FTD DPR proteins and VR/GL suggested that for these potentially toxic species to have been retained, one or both must have a valuable function in the cell cycle. Otherwise, over evolution, the mammalian (and *C. elegans*) telomeric repeats would have changed to ones that would not generate these DPRs. Of note, due to the random loading of ribosomes onto TERRA, one must assume that both VR and GL would be produced and thus it is not essential in this model that both would play some useful role.

**Figure 7.**
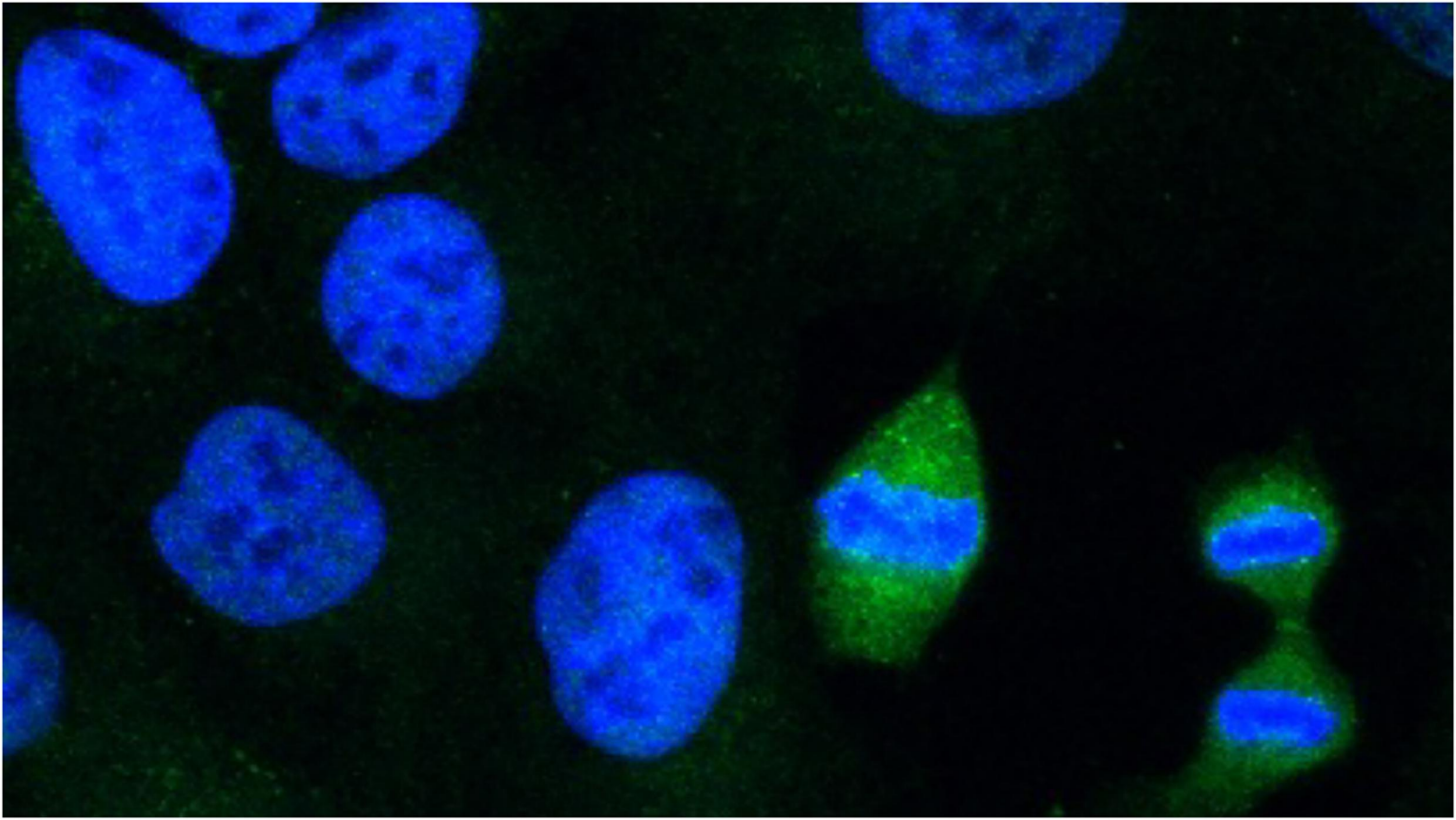
VR dipeptide repeat protein expressed in cultured mitotic cells, adapted from Al-Turki et al., 2025 [65]. Immunostaining for the VR dipeptide repeat protein (green) together with DAPI to mark DNA (blue) showed diffused VR signals in mitotic cells.

During a routine laser scanning confocal examination of cycling U2OS cells stained for VR using the polyclonal antibody, it was noted that in one field, two cells, which were in mitosis, stained brightly and diffusely, while the large number of surrounding cells, which were not dividing, showed only the small aggregates and speckles that were previously observed. Further examination revealed examples of cells in metaphase from primary foreskin cells and the classic 3T3 murine line, which also showed bright, diffuse VR staining in mitosis. Blocking U2OS cells in metaphase using the cdk1 inhibitor RO3306 followed by shake off of the mitotic cells and staining for VR revealed that 100% of the mitotic cells exhibited this bright diffuse staining across all stages of cell division [65]. An example is shown in our previously published work (Figure 7, [65]). Two possible models were considered based on these observations. In one, there would be a burst of new VR generated during cell division, due to the release of TERRA from the chromosomes and its engagement by cytoplasmic ribosomes. In the other model, VR is normally sequestered into aggregates and liquid droplets primarily in the nuclei of non-dividing cells; however, during cell division, the VR becomes dispersed, resulting in greater and diffuse antibody staining. In either case it was of interest to understand what role VR might be playing during mitosis.

Using cryoEM, Loveland et al. [31] showed that PR and GR bind to the exit tunnel in ribosomes. This suggested PR and GR may quench translation, and they demonstrated this using an *in vitro* firefly luciferase assay. Given the similarity of VR with GR and PR, we applied the same firefly luciferase assay and showed that VR inhibits translation with an efficiency that is only somewhat less than PR and GR [65]. This observation provided a possible role for VR in cell division: it has been known that global translation is reduced by 30–70% (depending on the assay) [83,84] during cell division, but the mechanisms behind this have not been fully elucidated, and thus the involvement of VR in this event was an attractive possibility.

If VR is involved in down-regulating translation, then one should see evidence for its interaction with the translation machinery using an IP and mass spectrometry approach. The VR antibody was attached to beads, incubated with U2OS cell extracts and the proteins bound to the beads via the VR antibody identified by mass spectrometry [65]. The results revealed ribosomal proteins including RL4 which lines the exit tunnel to be major binding targets along with proteins involved in the cell cycle and interestingly, numerous proteins with links to neurodegenerative diseases including ALS/FTD, Spinal Muscular Atrophies of Childhood, Multiple Sclerosis, SCA-17, ALS, Fragile X Tremor/Ataxia syndrome, Parkinson’s disease, Spinal Muscular Atrophy, Cerebellar Ataxia, Charcot–Marie–Tooth syndrome and Ataxia–telangiectasia-like syndrome and TBP-43 [65]. Table 1 provides a summary of some of these protein hits from this analysis. The presence of proteins involved in DNA replication may reflect the tight binding of VR observed *in vitro* and would underscore its possible role in errant replication events if VR was overproduced.

**Table 1.** Examples of proteins identified by IP/Mass spec as interactors with VR that are related to known neurological disease.

Protein IDs	Protein names	Gene names	Involved in
Q9H9T3	Elongator complex protein 3	ELP3	ALS/FTD
P35637	RNA-binding protein FUS	FUS	ALS/FTD
P28799	Granulins;Acrogranin;Paragranulin;Granulin-1;Granulin-2;Granulin-3;Granulin-4;Granulin-5;Granulin-6;Granulin-7	GRN	ALS/FTD
P09651; A0A2R8Y4L2; Q32P51	Heterogeneous nuclear ribonucleoprotein A1;Heterogeneous nuclear ribonucleoprotein A1, N-terminally processed; Heterogeneous nuclear ribonucleoprotein A1-like 2	HNRNPA1; HNRNPA1L2	ALS/FTD
P22626	Heterogeneous nuclear ribonucleoproteins A2/B1	HNRNPA2B1	ALS/FTD
P43243	Matrin-3	MATR3	ALS/FTD
Q13148	TAR DNA-binding protein 43	TARDBP	TDP-43 (TARDBP) | Disease: ALS/FTD
Q70J99	Protein unc-13 homolog D	UNC13D	ALS/FTD
Q14204	Cytoplasmic dynein 1 heavy chain 1	DYNC1H1	Spinal Muscular Atrophies of Childhood; Spinal Muscular Atrophy
Q9Y4W2	Ribosomal biogenesis protein LAS1L	LAS1L	Spinal Muscular Atrophies of Childhood; Spinal Muscular Atrophy
Q16637	Survival motor neuron protein	SMN1	Spinal Muscular Atrophies of Childhood; Spinal Muscular Atrophy
Q99986	Serine/threonine-protein kinase VRK1	VRK1	Spinal Muscular Atrophies of Childhood; Spinal Muscular Atrophy
P09543	2,3-cyclic-nucleotide 3-phosphodiesterase	CNP	Multiple Sclerosis
Q06787	Fragile X mental retardation protein 1	FMR1	Fragile X Tremor/Ataxia syndrome
Q04637	Eukaryotic translation initiation factor 4 gamma 1	EIF4G1	Parkinson disease
Q9Y4W6	AFG3-like protein 2	AFG3L2	Cerebellar Ataxia
Q99700	Ataxin-2	ATXN2	Cerebellar Ataxia
P26358	DNA (cytosine-5)-methyltransferase 1	DNMT1	Cerebellar Ataxia; Charcot-Marie-Tooth syndrome
O00567	Nucleolar protein 56	NOP56	Cerebellar Ataxia
Q96T60	Bifunctional polynucleotide phosphatase/kinase;Polynucleotide 3-phosphatase;Polynucleotide 5-hydroxyl-kinase	PNKP	Cerebellar Ataxia
P41250	Glycine-tRNA ligase	GARS	Charcot-Marie-Tooth syndrome
P04792	Heat shock protein beta-1	HSPB1	Charcot-Marie-Tooth syndrome
O60333	Kinesin-like protein KIF1B	KIF1B	Charcot-Marie-Tooth syndrome
Q92878	DNA repair protein RAD50	RAD50	Ataxia-telangiectasia-like syndrome

**Figure 8.**
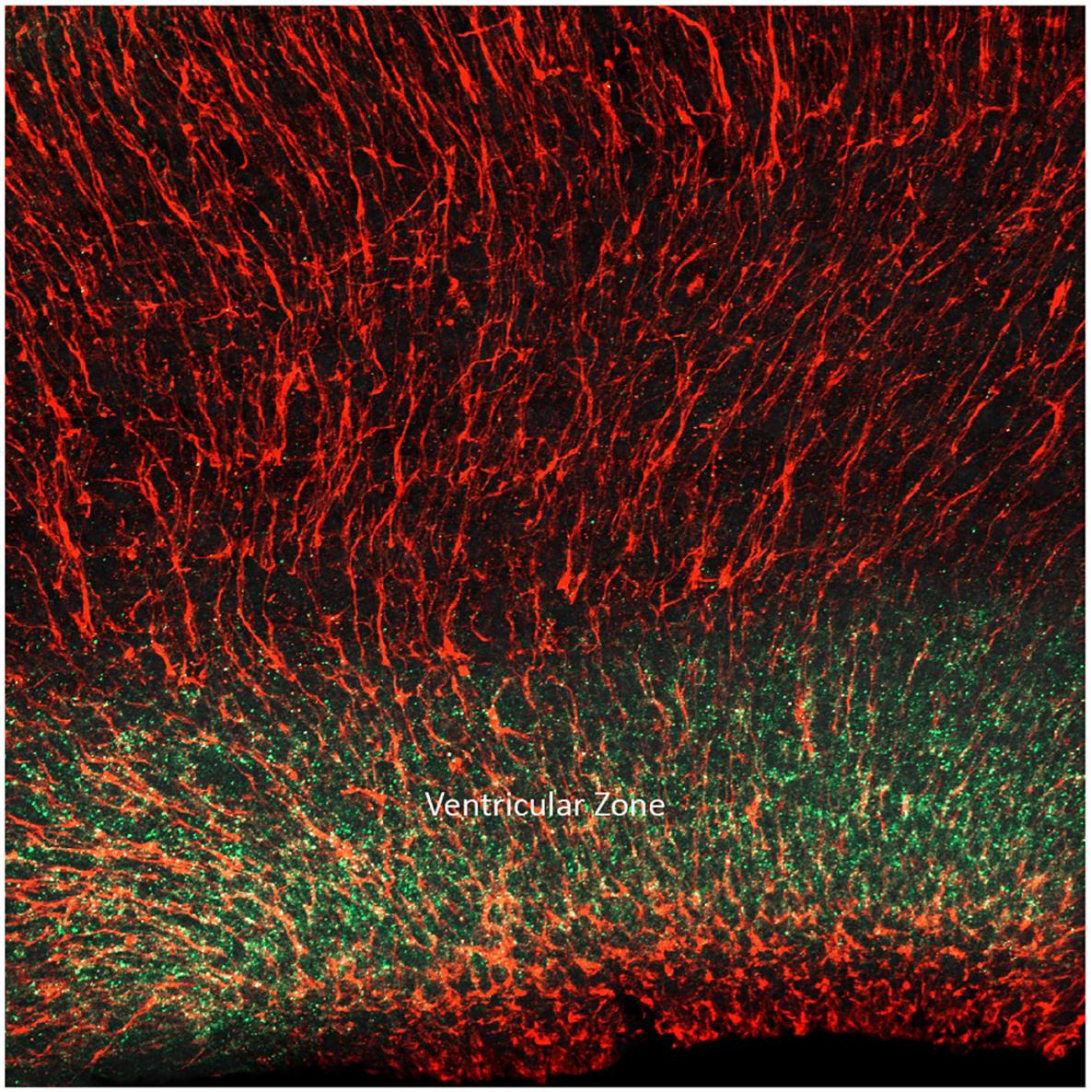
VR dipeptides repeat protein is expressed in proliferative progenitors of the developing mouse cerebral cortex. Representative coronal section of E16 mouse embryonic cortex immunolabeled for VR (anti-VR, green) and the cortical progenitor/radial glia marker RC2 (red). VR signal is prominent in the Ventricular Zone VZ, where neural progenitors proliferate.

The observation of bright diffuse VR staining in mitotic cells prompted an investigation of a mammalian tissue which might have a high mitotic index. The developing neural tube in mouse embryos at day 16 consists of a series of tissues beginning with the ventricular layer, in which cells are dividing very rapidly. Following division, cells migrate upward through the subventricular zone, intermediate zone and finally settle into the cortical plate [65]. Staining this tissue with the VR antibody showed very bright staining in the ventricular zone (Figure 8). In some examples, staining was also seen in the cortical plate, suggesting that the diffuse state of VR induced during cell division had remained with the cells during their migration to the cortical plate. While these studies have provided a first glimpse into possible role of VR in the cell, what mechanisms are involved in the cycle of VR synthesis and degradation remain to be understood.

## A model for the sequestration of the telomere DPRs in liquid phase bodies

Evidence suggests that VR possesses properties that could threaten cellular homoeostasis by binding nucleic acids (potentially including ribosomal RNA). In unstressed normal cells VR (and possibly GL) are not freely diffusible but rather accumulate in discrete nuclear puncta or droplet-like aggregates, suggesting that the cell actively sequesters VR (and likely GL) into phase-separated condensates. Confined to droplet-like aggregates, the ability of the highly charged VR species to bind nucleic acids and other essential cellular structures is minimized. Consequently, any telomere insult that would change the amount or location of TERRA in the cell will cause VR to redistribute from small aggregates into large, solid nuclear aggregates, an abnormal phase transition, suggestive of lost regulatory sequestration. This sequestration strategy provides cellular safeguard for managing aggregation-prone proteins, effectively neutralizing their harmful properties by isolating them in membraneless organelles. When telomere regulation fails, as in telomere uncapping, replication stress, this buffering system saturates, and excess VR peptides escape their phase-bound confines. In essence, the failure to maintain strict phase boundaries converts VR from inert structural peptides into pathogenic agents. The cellular consequences, such as inhibition of protein synthesis, nucleolar and replication stress, inflammation and DNA damage, mirror those observed when the C9orf72 DPRs overwhelm their normal sequestration. This model posits that the protective phase separation of telomeric VR peptides is crucial for cellular health, and that breaching these condensate barriers links telomere dysfunction to genomic instability.

## Could VR/GL provide a link between ageing and the formation of amyloid deposits in the brain?

As observed by EM, VR and GL assemble into rods and filaments ~10 nm in diameter, indistinguishable in appearance and dimensions to fibres formed by GA [7]. While the assembly process has not yet been established, presumably new VR or GL dipeptide molecules add onto the ends of existing filaments, much like recA filaments grow in a defined 5’ to 3’ direction on ssDNA [85]. At high concentrations, the filaments aggregate into amyloid mats typical, as seen by EM, of the amyloid deposits found in the brains of AD patients [7,65]. This suggested a possible link between ageing and development of AD.

**Table:** 

**Hypothesis: how GL/VR may seed Aβ amyloid plaque formation** As we age and our telomeres shorten, many reach the critical short length that results in the chain of events which we propose includes loss of the T-loop protection, telomere instability and greater production of VR and GL due release of TERRA from the chromosomes and its movement to the cytoplasm. This process would be amplified or appear sooner in age if our brain cells have been exposed to damaging toxic agents, reactive oxygen species or radiation. As the levels of VR and/or GL molecules in the brain cells increase, they will assemble into filaments which could provide molecular ends onto which free amyloid beta (Aβ) molecules could begin assembling into amyloid filaments. Aβ is known to form filaments with the same apparent structure as the VR/GL filaments and thus it is possible that VR or GL filaments could seed the formation of long Aβ filaments which would then generate amyloid plaques. Thus, we suggest that filament formation by Aβ is a slower process on its own but can be initiated earlier if seeded by the appearance of GL or VR filaments.

This model which could be tested via seeding experiments *in vitro*, is based on the knowledge that cells in the brain, including neurons, contain the amyloid precursor, which is processed to generate Aβ and the Aβ filaments have the same basic structure as ones formed by VR and GL. Since the prevailing view is that the Aβ plaques disrupt neuronal function, resulting in AD, one must ask, “If amyloid precursor protein is present from childhood, why does it take so many years for the aggregates to appear? This model would suggest that indeed Aβ aggregation slowly occurs as we age, but if VR and/or GL filaments appear in brain cells earlier in life due to telomere damage, this can stimulate amyloid formation by providing filament ends onto which Aβ can initiate assembly leading to large amyloid deposits.

In a preliminary experiment conducted in collaboration with Todd Cohen’s laboratory at UNC, VR staining was observed to colocalize with a subset of amyloid plaque signals in brain tissue from an Alzheimer’s patient, although the degree of overlap varied across plaques (full, partial or absent). This might suggest that VR or GL assemblies could participate in, or be recruited to, amyloid-rich microenvironments. Future work should test this directly using validated specificity controls and quantitative co-localization analysis, along with *in vitro* cross-seeding assays to test whether VR/GL filaments nucleate or accelerate amyloid formation.

## Can VR and GL be carried as cargo in extracellular vesicles?

The IP/mass spec analysis (Table 1) identified several exosome structural proteins (ANXA2, ARF6, RAB11A/RAB11B, HSPA8 and HSP90AB1) as significant VR interactors. It will be important to determine whether VR and or GL are carried as cargo in extracellular vesicles (EVs or exosomes). If so, their dispersal could provide a mechanism (based on the model above) by which the formation of pathological amyloid deposits could spread rapidly across the brain once a threshold of VR or GL concentrations had been achieved. Such an effect could be further amplified if the uptake of VR or GL into cells were to trigger toxic pathways, which themselves would result in further production or release of VR/GL.

## Can VR/GL serve as biomarkers for toxic insult, ageing and disease?

In acute exposure and toxicology settings (e.g. ionizing radiation, topoisomerase poisons, oxidative stressors), transient rises in VR would be expected to mirror bursts of damage factors and p53 activation and could serve as a post-chemo/radiotherapy marker (pharmacodynamic marker) of telomere distress. In gerontological science, chronic low-grade replication and telomere stress may yield a basal VR signal that tracks biological ageing more specifically than broad inflammatory markers and with faster kinetics than telomere length. Disease applications include neurodegeneration, where VR could reflect neuronal stress and glial innate activation, and telomere biology disorders such as dyskeratosis congenita, bone-marrow failure or short-telomere-associated pulmonary fibrosis, in which VR would be a mechanism-linked activity marker. In oncology, telomere crisis, ALT activity and DNA-damaging therapies could generate treatment-responsive VR signals useful for target engagement and dose optimization. Episodes of extreme physiology, including spaceflight, represent additional scenarios in which VR may report situational exposure and recovery.

Currently, telomere length measurement (TLM) provides the only measure of telomere integrity. This measurement, however, is severely complicated by the fact that it remains unclear whether the most important feature is the length of the shortest telomeres in the population, the longest or some mean. TLM also reflects the result of long-term erosion of telomeres rather than the overall health of our telomeres at the time of measurement. Once fully developed, a simple clinical measurement of the level of VR or GL in plasma will provide a measure of current telomere health and a single numerical value which can be compared across broad studies. It will thus inform on chronological age in healthy individuals and raise a flag for the presence of disease, particularly due to disorders driven by primary telomere dysfunction.

## Summary

The RAN mechanism first identified in studies of rare neurological diseases likely plays important roles in many normal cellular processes far beyond the pathological settings where it was first discovered. The similarity of the repetitive RNAs central to ALS/FTD and to telomeres led to our demonstration of two RAN products, VR and GL produced from mammalian telomeres, both of which show strong structural parallels with the 5 RAN products of the ALS/FTD transcripts. These parallels have helped point the way to uncovering biological properties of VR and GL. In particular, the observation that VR undergoes a change in its cellular distribution during mitosis and inhibits translation by ribosomes highlights a link between telomeres and cell cycle regulation. These observations suggest that the highly conserved telomeric TTAGGG repeat motif has been retained in evolution for chromosome end protection and for the production of regulatory peptides. Consistent with this view, it was observed that VR is expressed in the murine embryonic neural progenitor zone where cells are undergoing rapid division, implying a role for telomere-encoded peptides in coordinating mitotic progression. While the physical and biological properties of both VR and GL have only begun to be elucidated, the high affinity of VR for nucleic acids and self-assembly of VR and GL into amyloid-like filaments have obvious ramifications for DNA damage response and for the possible link between ageing, DNA damage, and the development of amyloid plaques. This link is further supported by the studies showing that the GR and PR DPRs are implicated in ALS/FTD neurodegeneration.

Despite this progress, several foundational questions remain. First, the cellular properties and functions of GL are largely undefined, due to lack of a validated GL-specific antibody. The half-life of these DPRs in the cell is unknown. It also remains unknown whether mixed VR/GL dipeptide-repeat species exist *in vivo* and whether the VR antibodies partially detect such mixed assemblies. High-resolution structural information is lacking for VR/GL filaments, including how filament architecture depends on repeat length and whether structural transitions accompany oligomer to fibril conversion. Finally, the molecular composition of VR-containing phase-separated bodies remains incompletely characterized, hindering our understanding of how these assemblies form and how they impact cellular functions.

## Data Availability

The data supporting Figure 3 in this article are openly available in Figshare at https://doi.org/10.6084/m9.figshare.32024601.

## References

[1] Zu T, Gibbens B, Doty NS, et al. Non-ATG-initiated translation directed by microsatellite expansions. Proc Natl Acad Sci USA. 2011;108(1):260–265. doi: 10.1073/pnas.1013343108 21173221 PMC3017129

[2] DeJesus-Hernandez M, Mackenzie IR, Boeve BF, et al. Expanded GGGGCC hexanucleotide repeat in noncoding region of C9ORF72 causes chromosome 9p-linked FTD and ALS. Neuron. 2011;72(2). doi: 10.1016/j.neuron.2011.09.011 PMC3202986 21944778

[3] Renton AE, Majounie E, Waite A, et al. A hexanucleotide repeat expansion in C9ORF72 is the cause of chromosome 9p21-linked ALS-FTD. Neuron. 2011;72(2):257–268. doi: 10.1016/j.neuron.2011.09.010 21944779 PMC3200438

[4] Boeynaems S, Bogaert E, Kovacs D, et al. Phase separation of C9orf72 dipeptide repeats perturbs stress granule dynamics. Mol Cell. 2017;65(6):1044–1055.e5. doi: 10.1016/j.molcel.2017.02.013 28306503 PMC5364369

[5] Azzalin CM, Reichenbach P, Khoriauli L, et al. Telomeric repeat-containing RNA and RNA surveillance factors at mammalian chromosome ends. Science. 2007;318(5851):798–801. doi: 10.1126/science.1147182 17916692

[6] Schoeftner S, Blasco MA. Developmentally regulated transcription of mammalian telomeres by DNA-dependent RNA polymerase II. Nat Cell Biol. 2008;10(2):228–236. doi: 10.1038/ncb1685 18157120

[7] Al-Turki TM, Griffith JD. Mammalian telomeric RNA (TERRA) can be translated to produce valine–arginine and glycine–leucine dipeptide repeat proteins. Proc Natl Acad Sci USA. 2023;120(9). doi: 10.1073/pnas.2221529120 PMC9992779 36812212

[8] Kearse MG, Goldman DH, Choi J, et al. Ribosome queuing enables non-AUG translation to be resistant to multiple protein synthesis inhibitors. Genes Dev. 2019;33(13–14):871–885. doi: 10.1101/gad.324715.119 31171704 PMC6601509

[9] Green KM, Glineburg MR, Kearse MG, et al. RAN translation at C9orf72-associated repeat expansions is selectively enhanced by the integrated stress response. Nat Commun. 2017;8(1):2005. doi: 10.1038/s41467-017-02200-0 29222490 PMC5722904

[10] Cleary JD, Ranum LPW. Repeat-associated non-ATG (RAN) translation in neurological disease. Hum Mol Genet. 2013;22(R1):R45–R51. doi: 10.1093/hmg/ddt371 23918658 PMC3782068

[11] Tabet R, Schaeffer L, Freyermuth F, et al. CUG initiation and frameshifting enable production of dipeptide repeat proteins from ALS/FTD C9ORF72 transcripts. Nat Commun. 2018;9(1):152. doi: 10.1038/s41467-017-02643-5 29323119 PMC5764992

[12] Fujino Y, Mori K, Nagai Y. Repeat-associated non-AUG translation in neuromuscular diseases: mechanisms and therapeutic insights. J Biochem. 2023;173(4):273–281. doi: 10.1093/jb/mvad012 36748359

[13] Sellier C, Buijsen RAM, He F, et al. Translation of expanded CGG repeats into FMRpolyG is pathogenic and may contribute to fragile X tremor ataxia syndrome. Neuron. 2017;93(2):331–347. doi: 10.1016/j.neuron.2016.12.016 28065649 PMC5263258

[14] Kearse MG, Green KM, Krans A, et al. CGG repeat-associated non-AUG translation utilizes a cap-dependent scanning mechanism of initiation to produce toxic proteins. Mol Cell. 2016;62(2):314–322. doi: 10.1016/j.molcel.2016.02.034 27041225 PMC4854189

[15] Cheng W, Wang S, Mestre AA, et al. C9ORF72 GGGGCC repeat-associated non-AUG translation is upregulated by stress through eIf2α phosphorylation. Nat Commun. 2018;9(1):51. doi: 10.1038/s41467-017-02495-z 29302060 PMC5754368

[16] Zu T, Guo S, Bardhi O, et al. Metformin inhibits RAN translation through PKR pathway and mitigates disease in *C9orf72* ALS/FTD mice. Proc Natl Acad Sci USA. 2020;117(31):18591–18599. doi: 10.1073/pnas.2005748117 32690681 PMC7414156

[17] Green KM, Miller SL, Malik I, et al. Non-canonical initiation factors modulate repeat-associated non-AUG translation. Hum Mol Genet. 2022;31(15):2521–2534. doi: 10.1093/hmg/ddac021 35220421 PMC9618161

[18] May S, Hornburg D, Schludi MH, et al. C9orf72 FTLD/ALS-associated Gly-Ala dipeptide repeat proteins cause neuronal toxicity and Unc119 sequestration. Acta Neuropathol. 2014;128(4):485–503. doi: 10.1007/s00401-014-1329-4 25120191 PMC4159571

[19] Yamakawa M, Ito D, Honda T, et al. Characterization of the dipeptide repeat protein in the molecular pathogenesis of c9FTD/ALS. Hum Mol Genet. 2015;24(6):1630–1645. doi: 10.1093/hmg/ddu576 25398948

[20] Zhang YJ, Jansen-West K, Xu YF, et al. Aggregation-prone c9FTD/ALS poly(GA) RAN-translated proteins cause neurotoxicity by inducing ER stress. Acta Neuropathol. 2014;128(4):505–524. doi: 10.1007/s00401-014-1336-5 25173361 PMC4159567

[21] Choi SY, Lopez-Gonzalez R, Krishnan G, et al. C9ORF72-ALS/FTD-associated poly(GR) binds Atp5a1 and compromises mitochondrial function in vivo. Nat Neurosci. 2019;22(6):851–862. doi: 10.1038/s41593-019-0397-0 31086314 PMC6800116

[22] Freibaum BD, Lu Y, Lopez-Gonzalez R, et al. GGGGCC repeat expansion in C9orf72 compromises nucleocytoplasmic transport. Nature. 2015;525(7567):129–133. doi: 10.1038/nature14974 26308899 PMC4631399

[23] Jovičič A, Mertens J, Boeynaems S, et al. Modifiers of C9orf72 dipeptide repeat toxicity connect nucleocytoplasmic transport defects to FTD/ALS. Nat Neurosci. 2015;18(9):1226–1229. doi: 10.1038/nn.4085 26308983 PMC4552077

[24] Lopez-Gonzalez R, Lu Y, Gendron TF, et al. Poly(GR) in C9ORF72-related ALS/FTD compromises mitochondrial function and increases oxidative stress and DNA damage in iPSC-derived motor neurons. Neuron. 2016;92(2):383–391. doi: 10.1016/j.neuron.2016.09.015 27720481 PMC5111366

[25] Park J, Lee J, Kim JH, et al. ZNF598 co-translationally titrates poly(GR) protein implicated in the pathogenesis of C9ORF72-associated ALS/FTD. Nucleic Acids Res. 2021;49(19):11294–11311. doi: 10.1093/nar/gkab834 34551427 PMC8565315

[26] Radwan M, Ang CS, Ormsby AR, et al. Arginine in C9ORF72 dipolypeptides mediates promiscuous proteome binding and multiple modes of toxicity. Mol Cellular Proteomics. 2020;19(4):640–654. doi: 10.1074/mcp.RA119.001888 PMC7124463 32086375

[27] Xinmei W, Wenzhi T, Thomas W, et al. Antisense proline-arginine RAN dipeptides linked to C9ORF72- ALS/FTD form toxic nuclear aggregates that initiate in vitro and in vivo neuronal death. Neuron. 2014;84(6):1213–1225. doi: 10.1016/j.neuron.2014.12.010 25521377 PMC4632245

[28] Zhang YJ, Gendron TF, Ebbert MTW, et al. Poly(GR) impairs protein translation and stress granule dynamics in C9orf72-associated frontotemporal dementia and amyotrophic lateral sclerosis. Nat Med. 2018;24(8):1136–1142. doi: 10.1038/s41591-018-0071-1 29942091 PMC6520050

[29] Zhang YJ, Guo L, Gonzales PK, et al. Heterochromatin anomalies and double-stranded RNA accumulation underlie C9orf72 poly(PR) toxicity. Science. 2019;363(6428). doi: 10.1126/science.aav2606 PMC6524780 30765536

[30] Kwon I, Xiang S, Kato M, et al. Poly-dipeptides encoded by the C9orf72 repeats bind nucleoli, impede RNA biogenesis, and kill cells. Science. 2014;345(6201):1139–1145. doi: 10.1126/science.1254917 25081482 PMC4459787

[31] Loveland AB, Svidritskiy E, Susorov D, et al. Ribosome inhibition by C9ORF72-ALS/FTD-associated poly-PR and poly-GR proteins revealed by cryo-EM. Nat Commun. 2022;13(1). doi: 10.1038/s41467-022-30418-0 PMC9120013 35589706

[32] Chew J, Gendron TF, Prudencio M, et al. C9ORF72 repeat expansions in mice cause TDP-43 pathology, neuronal loss, and behavioral deficits. Science. 2015;348(6239). doi: 10.1126/science.aaa9344 PMC4692360 25977373

[33] Liu Y, Pattamatta A, Zu T, et al. C9orf72 BAC mouse model with motor deficits and neurodegenerative features of ALS/FTD. Neuron. 2016;90(3):521–534. doi: 10.1016/j.neuron.2016.04.005 27112499

[34] Nguyen L, Montrasio F, Pattamatta A, et al. Antibody therapy targeting RAN proteins rescues C9 ALS/FTD phenotypes in C9orf72 mouse model. Neuron. 2020;105(4):645–662.e11. doi: 10.1016/j.neuron.2019.11.007 31831332 PMC7391607

[35] Jiang X, Schaeffer L, Patni D, et al. Blocking RAN translation without altering repeat RNAs rescues *C9ORF72* -related ALS and FTD phenotypes. Science. 2026;391(6785):eadv2600. doi: 10.1126/science.adv2600 41643021 PMC13107528

[36] Shaw MP, Higginbottom A, McGown A, et al. Stable transgenic C9orf72 zebrafish model key aspects of the ALS/FTD phenotype and reveal novel pathological features. Acta Neuropathol Commun. 2018;6(1). doi: 10.1186/s40478-018-0629-7 PMC6240957 30454072

[37] Swinnen B, Bento-Abreu A, Gendron TF, et al. A zebrafish model for C9orf72 ALS reveals RNA toxicity as a pathogenic mechanism. Acta Neuropathol. 2018;135(3):427–443. doi: 10.1007/s00401-017-1796-5 29302778

[38] O’Rourke JG, Bogdanik L, Muhammad AKMG, et al. C9orf72 BAC transgenic mice display typical pathologic features of ALS/FTD. Neuron. 2015;88(5):892–901. doi: 10.1016/j.neuron.2015.10.027 26637796 PMC4672384

[39] Brangwynne CP, Eckmann CR, Courson DS, et al. Germline P granules are liquid droplets that localize by controlled dissolution/condensation. Science. 2009;324(5935):1729–1732. doi: 10.1126/science.1172046 19460965

[40] Jafarinia H, van der Giessen E, Onck PR. Phase separation of toxic dipeptide repeat proteins related to C9orf72 ALS/FTD. Biophys J. 2020;119(4):843–851. doi: 10.1016/j.bpj.2020.07.005 32730793 PMC7451925

[41] Gittings LM, Boeynaems S, Lightwood D, et al. Symmetric dimethylation of poly-GR correlates with disease duration in C9orf72 FTLD and ALS and reduces poly-GR phase separation and toxicity. Acta Neuropathol. 2020;139(2):407–410. doi: 10.1007/s00401-019-02104-x 31832771 PMC6989575

[42] Chen C, Yamanaka Y, Ueda K, et al. Phase separation and toxicity of c9orf72 poly(pr) depends on alternate distribution of arginine. J Cell Bio. 2021;220(11). doi: 10.1083/jcb.202103160 PMC8438627 34499080

[43] White MR, Mitrea DM, Zhang P, et al. C9orf72 Poly(PR) dipeptide repeats disturb biomolecular phase separation and disrupt nucleolar function. Mol Cell. 2019;74(4):713–728.e6. doi: 10.1016/j.molcel.2019.03.019 30981631 PMC6525025

[44] Patel A, Lee HO, Jawerth L, et al. A liquid-to-solid phase transition of the ALS protein FUS accelerated by disease mutation. Cell. 2015;162(5):1066–1077. doi: 10.1016/j.cell.2015.07.047 26317470

[45] Olovnikov AM. A theory of marginotomy. The incomplete copying of template margin in enzymic synthesis of polynucleotides and biological significance of the phenomenon. J Theor Biol. 1973;41(1):181–190. doi: 10.1016/0022-5193(73)90198-7 PubMed PMID: 4754905. 4754905

[46] Griffith JD, Comeau L, Rosenfield S, et al. Mammalian telomeres end in a large duplex loop. Cell. 1999;97(4):503–514. doi: 10.1016/S0092-8674(00)80760-6 10338214

[47] Romero-Zamora D, Rogers S, Low RRJ, et al. A CPC-shelterin-BTR axis regulates mitotic telomere deprotection. Nat Commun. 2025;16(1):2277. doi: 10.1038/s41467-025-57456-8 40097392 PMC11914695

[48] Tomáška, Cesare AJ, AlTurki TM, et al. Twenty years of t-loops: a case study for the importance of collaboration in molecular biology. DNA Repair (Amst). 2020;94:94. doi: 10.1016/j.dnarep.2020.102901 PMC8679138 32620538

[49] Doksani Y. The response to dna damage at telomeric repeats and its consequences for telomere function. Genes (Basel). 2019;10(4):318. doi: 10.3390/genes10040318 31022960 PMC6523756

[50] Watson JD. Origin of concatemeric T7 DNA. Nat New Biol. 1972;239(94):197–201. doi: 10.1038/newbio239197a0 PubMed PMID: 4507727. 4507727

[51] D’Alessandro G, Whelan DR, Howard SM, et al. BRCA2 controls DNA: RNA hybrid level at DSBs by mediating RNase H2 recruitment. Nat Commun. 2018;9(1):5376. doi: 10.1038/s41467-018-07799-2 30560944 PMC6299093

[52] Harley CB, Futcher AB, Greider CW. Telomeres shorten during ageing of human fibroblasts. Nature. 1990;345(6274):458–460. doi: 10.1038/345458a0 PubMed PMID: 2342578. 2342578

[53] Allsopp RC, Chang E, Kashefi-Aazam M, et al. Telomere shortening is associated with cell division in vitro and in vivo. Exp Cell Res. 1995;220(1):194–200. doi: 10.1006/excr.1995.1306 PubMed PMID: 7664836. 7664836

[54] Feuerhahn S, Iglesias N, Panza A, et al. TERRA biogenesis, turnover and implications for function. FEBS Lett. 2010;584(17):3812–3818. doi: 10.1016/j.febslet.2010.07.032 20655916

[55] Porro A, Feuerhahn S, Reichenbach P, et al. Molecular dissection of telomeric repeat-containing RNA biogenesis unveils the presence of distinct and multiple regulatory pathways. Mol Cell Biol. 2010;30(20):4808–4817. doi: 10.1128/mcb.00460-10 20713443 PMC2950545

[56] Savoca V, Rivosecchi J, Gaiatto A, et al. TERRA stability is regulated by RALY and polyadenylation in a telomere-specific manner. Cell Rep. 2023;42(4):112406. doi: 10.1016/j.celrep.2023.112406 37060569

[57] Larini L, Goretti E, Zulian E, et al. Telomeric lncRNA TERRA localizes to stress granules in human ALT cells [internet]. Mol Biol. 2024. doi: 10.1101/2024.06.18.599513

[58] Fay MM, Anderson PJ, Ivanov P. ALS/FTD-Associated C9ORF72 repeat RNA promotes phase transitions in vitro and in cells. Cell Rep. 2017;21(12):3573–3584. doi: 10.1016/j.celrep.2017.11.093 29262335 PMC5741101

[59] Rossi S, Serrano A, Gerbino V, et al. Nuclear accumulation of mRNAs underlies G4C2 repeat-induced translational repression in a cellular model of *C9orf72* ALS. J Cell Sci. 2015:jcs.165332. doi: 10.1242/jcs.165332 25788698

[60] Lin SJ, Rosas E, Fuchs G, et al. Lost in translation: when the rules do not apply. WIREs RNA. 2025;16(6):e70029. doi: 10.1002/wrna.70029 41224723 PMC13487876

[61] Galigniana NM, Charó NL, Uranga R, et al. Oxidative stress induces transcription of telomeric repeat-containing RNA (TERRA) by engaging PKA signaling and cytoskeleton dynamics. Biochim Biophys Acta (BBA) - Mol Cell Res. 2020;1867(4):118643. doi: 10.1016/j.bbamcr.2020.118643 31917282

[62] Porro A, Feuerhahn S, Delafontaine J, et al. Functional characterization of the TERRA transcriptome at damaged telomeres. Nat Commun. 2014;5(1):5379. doi: 10.1038/ncomms6379 25359189 PMC4264578

[63] Nassour J, Aguiar LG, Correia A, et al. Telomere-to-mitochondria signalling by ZBP1 mediates replicative crisis. Nature. 2023;614(7949):767–773. doi: 10.1038/s41586-023-05710-8 36755096 PMC9946831

[64] Nassour J, Przetocka S, Karlseder J. Telomeres as hotspots for innate immunity and inflammation. DNA Repair (Amst). 2024;133:103591. doi: 10.1016/j.dnarep.2023.103591 37951043 PMC10842095

[65] Al-Turki T, Mantri V, Willcox S, et al. The telomeric valine–arginine dipeptide repeat protein changes state to diffuse staining in mitosis and represses in vitro translation. Proc Natl Acad Sci USA. 2025;122(47):e2520441122. doi: 10.1073/pnas.2520441122 41269794 PMC12663981

[66] Tseng YJ, Sandwith SN, Green KM, et al. The RNA helicase DHX36–G4R1 modulates C9orf72 GGGGCC hexanucleotide repeat–associated translation. J Biol Chem. 2021;297(2):100914. doi: 10.1016/j.jbc.2021.100914 34174288 PMC8326427

[67] Sexton AN, Collins K. The 5′ guanosine tracts of human telomerase RNA are recognized by the G-Quadruplex binding Domain of the RNA Helicase DHX36 and function to increase RNA accumulation. Mol Cell Biol. 2011;31(4):736–743. doi: 10.1128/MCB.01033-10 21149580 PMC3028649

[68] Chang YJ, Jeng US, Chiang YL, et al. The glycine-alanine dipeptide repeat from C9 or f72 hexanucleotide expansions forms toxic amyloids possessing cell-to-cell transmission properties. J Biol Chem. 2016;291(10). doi: 10.1074/jbc.M115.694273 PMC4777828 26769963

[69] Flores BN, Dulchavsky ME, Krans A, et al. Distinct c9orf72-associated dipeptide repeat structures correlate with neuronal toxicity. PLOS ONE. 2016;11(10):e0165084. doi: 10.1371/journal.pone.0165084 27776165 PMC5077081

[70] Marques C, Held A, Dorfman K, et al. Neuronal STING activation in amyotrophic lateral sclerosis and frontotemporal dementia. Acta Neuropathol. 2024;147(1):56. doi: 10.1007/s00401-024-02688-z 38478117 PMC10937762

[71] Fu RH, Tsai CW, Chiu SC, et al. C9-ALS-Associated proline-arginine dipeptide repeat protein induces activation of NLRP3 inflammasome of HMC3 microglia cells by binding of complement component 1 Q subcomponent-binding protein (C1QBP), and syringin prevents this effect. Cells. 2022;11(19):3128. doi: 10.3390/cells11193128 36231090 PMC9563448

[72] Fu RH, Chen HJ, Hong SY. Glycine-alanine dipeptide repeat protein from C9-ALS interacts with sulfide quinone oxidoreductase (SQOR) to induce the activity of the NLRP3 inflammasome in HMC3 microglia: irisflorentin reverses this interaction. Antioxidants. 2023;12(10):1896. doi: 10.3390/antiox12101896 37891975 PMC10604625

[73] Arora R, Lee Y, Wischnewski H, et al. RNaseH1 regulates TERRA-telomeric DNA hybrids and telomere maintenance in ALT tumour cells. Nat Commun. 2014;5(1):5. doi: 10.1038/ncomms6220 PMC4218956 25330849

[74] Ng LJ, Cropley JE, Pickett HA, et al. Telomerase activity is associated with an increase in DNA methylation at the proximal subtelomere and a reduction in telomeric transcription. Nucleic Acids Res. 2009;37(4):1152–1159. doi: 10.1093/nar/gkn1030 19129228 PMC2651807

[75] Sagie S, Toubiana S, Hartono SR, et al. Telomeres in ICF syndrome cells are vulnerable to DNA damage due to elevated DNA:RNA hybrids. Nat Commun. 2017;8(1):8. doi: 10.1038/ncomms14015 28117327 PMC5286223

[76] Yehezkel S, Segev Y, Viegas-Péquignot E, et al. Hypomethylation of subtelomeric regions in ICF syndrome is associated with abnormally short telomeres and enhanced transcription from telomeric regions. Hum Mol Genet. 2008;17(18):2776–2789. doi: 10.1093/hmg/ddn177 18558631

[77] González-Vasconcellos I, Cobos-Fernández MA, Atkinson MJ, et al. Quantifying telomeric lncRnas using PNA-labelled RNA-Flow FISH (RNA-Flow). Commun Biol. 2022;5(1):513. doi: 10.1038/s42003-022-03452-3 PubMed PMID: 35614335. 35614335 PMC9132901

[78] Cesare AJ, Hayashi MT, Crabbe L, et al. The telomere deprotection response is functionally distinct from the genomic DNA damage response. Mol Cell. 2013;51(2):141–155. doi: 10.1016/j.molcel.2013.06.006 23850488 PMC3721072

[79] Burger AM, Dai F, Schultes CM, et al. The G-quadruplex-interactive molecule BRACO-19 inhibits tumor growth, consistent with telomere targeting and interference with telomerase function. Cancer Res. 2005;65(4):1489–1496. doi: 10.1158/0008-5472.CAN-04-2910 15735037

[80] Bidzinska J, Di Pietro L, Naghshineh E, et al. Cellular adaptations impact the biological activity of naphthalene diimide G-quadruplex ligands in ALT-positive osteosarcoma cells. Cell Death Dis. 2025 ;16(1):581. doi: 10.1038/s41419-025-07908-2 40750583 PMC12316980

[81] Fumagalli L, Young FL, Boeynaems S, et al. C9orf72-derived arginine-containing dipeptide repeats associate with axonal transport machinery and impede microtubule-based motility. Sci Adv. 2021;7(15). doi: 10.1126/SCIADV.ABG3013 PMC8034861 33837088

[82] Hayes LR, Duan L, Bowen K, et al. C9orf72 arginine-rich dipeptide repeat proteins disrupt karyopherin-mediated nuclear import. Elife. 2020;9. doi: 10.7554/eLife.51685 PMC7051184 32119645

[83] Fan H, Penman S. Regulation of protein synthesis in mammalian cells. II. Inhibition of protein synthesis at the level of initiation during mitosis. J Mol Biol. 1970;50(3):655–670. doi: 10.1016/0022-2836(70)90091-4 5529301

[84] Tanenbaum ME, Stern-Ginossar N, Weissman JS, et al. Regulation of mRNA translation during mitosis. Elife. 2015;4(AUGUST2015). doi: 10.7554/eLife.07957 PMC4548207 26305499

[85] Register JC, Griffith J. The direction of RecA protein assembly onto single strand DNA is the same as the direction of strand assimilation during strand exchange. J Biol Chem. 1985;260(22):12308–12312. doi: 10.1016/S0021-9258(17)39026-9 PubMed PMID: 3900072. 3900072

